# Behavioral Mechanism during Human Sperm Chemotaxis: Involvement of Hyperactivation

**DOI:** 10.1371/journal.pone.0028359

**Published:** 2011-12-07

**Authors:** Leah Armon, Michael Eisenbach

**Affiliations:** Department of Biological Chemistry, The Weizmann Institute of Science, Rehovot, Israel; University of Hull, United Kingdom

## Abstract

When mammalian spermatozoa become capacitated they acquire, among other activities, chemotactic responsiveness and the ability to exhibit occasional events of hyperactivated motility—a vigorous motility type with large amplitudes of head displacement. Although a number of roles have been proposed for this type of motility, its function is still obscure. Here we provide evidence suggesting that hyperactivation is part of the chemotactic response. By analyzing tracks of spermatozoa swimming in a spatial chemoattractant gradient we demonstrate that, in such a gradient, the level of hyperactivation events is significantly lower than in proper controls. This suggests that upon sensing an increase in the chemoattractant concentration capacitated cells repress their hyperactivation events and thus maintain their course of swimming toward the chemoattractant. Furthermore, in response to a temporal concentration jump achieved by photorelease of the chemoattractant progesterone from its caged form, the responsive cells exhibited a delayed turn, often accompanied by hyperactivation events or an even more intense response in the form of flagellar arrest. This study suggests that the function of hyperactivation is to cause a rather sharp turn during the chemotactic response of capacitated cells so as to assist them to reorient according to the chemoattractant gradient. On the basis of these results a model for the behavior of spermatozoa responding to a spatial chemoattractant gradient is proposed.

## Introduction

Within the female genital tract mammalian spermatozoa are known to undergo a process of maturation, termed capacitation, which confers on them a number of capabilities: the ability to be released from the sperm storage site in the isthmic part of the Fallopian tube, to be guided by thermotaxis and chemotaxis (thought to serve as long-range and short-range guidance mechanisms, respectively), to penetrate the cumulus layers surrounding the oocyte, to bind to the oocyte, and to undergo the acrosome reaction that enables oocyte penetration [1], [2]. Two important facts about capacitation are that the fraction of capacitated cells is small at any given moment and that the capacitated state is short-lived (1–4 h in humans *in vitro*) [3]. These facts, the small number of spermatozoa that reach the Fallopian tube, and the relatively long way that spermatozoa have to do in the Fallopian tube before they can reach the oocyte suggest that guidance is essential for sperm arrival to the oocyte [2].

Another activity restricted to capacitated spermatozoa is hyperactivated motility. Unlike normal motility — swimming in rather straight lines, hyperactivated motility is non-linear, characterized by increased velocity, large amplitude of lateral head displacement, and intense flagellar whiplash movements [4], reflected in vigorous movements from side to side. Hyperactivation is initiated by elevation of intracellular Ca^2+^ (Ca^2+^_in_), probably via Ca^2+^ influx through the CatSper Ca^2+^ channel [5], although the involvement of intracellular Ca^2+^ stores, such as the redundant nuclear envelope store, was suggested as well [6]. The CatSper channel was identified in mammals and found to be specific for male germ cells [7], [8], [9]. The prominent phenotype of knockout mice missing any one of the four proteins that constitute the CatSper channel is that they are infertile and their spermatozoa do not become hyperactivated [10]. Also, these proteins were shown to be essential for human fertility [11], [12], [13]. Recently, human CatSper (or a protein closely associated with it) was demonstrated to be the chemoreceptor for the sperm chemoattractant progesterone [14], [15].

Hyperactivated motility appears crucial for fertilization: it was proposed to be involved in the detachment of capacitated spermatozoa from the sperm reservoir at the isthmic epithelium [16], [17], to assist spermatozoa to move through the viscous environment of the oviduct [18], and to penetrate the zona pellucida [19] (though only in the latter case [19] a reliable distinction between capacitated and hyperactivated cells was made). The observations that the processes of sperm hyperactivation and taxis (both thermotaxis and chemotaxis) are each restricted to capacitated cells only [3], [20], [21], that both sperm chemotaxis [22], [23] and hyperactivation [24] require extracellular Ca^2+^, that the episodes of hyperactivated motility are transient [20], and that hyperactivation (just like tumbling of bacteria such as *Escherichia coli* [25]) may result in a change in the swimming direction, led us to raise the possibility that, like in the case of bacterial chemotaxis, hyperactivation may be a mechanism of changing the direction of swimming during taxis [22]. The aim of this undertaking was to examine this possibility.

## Results

A direct way to determine the involvement of hyperactivation in the chemotactic response would be specific inhibition of hyperactivation and examining its effect on chemotaxis. But, regretfully, such specific inhibitors are currently unknown. Moreover, even inhibition of molecular processes known to be involved in hyperactivation (e.g., Ca^2+^_in_ elevation [5]) is impossible because these processes are also involved in chemotaxis (e.g., [2], [23], [26]). An exception could have been inhibition of the CatSper channel. However, the recently suggested possibility that this channel (or a protein closely associated with it) is the receptor for the chemoattractant progesterone [14], [15] excludes the inhibition of CatSper as a means of distinction between hyperactivation and chemotaxis.

**Figure 1:**
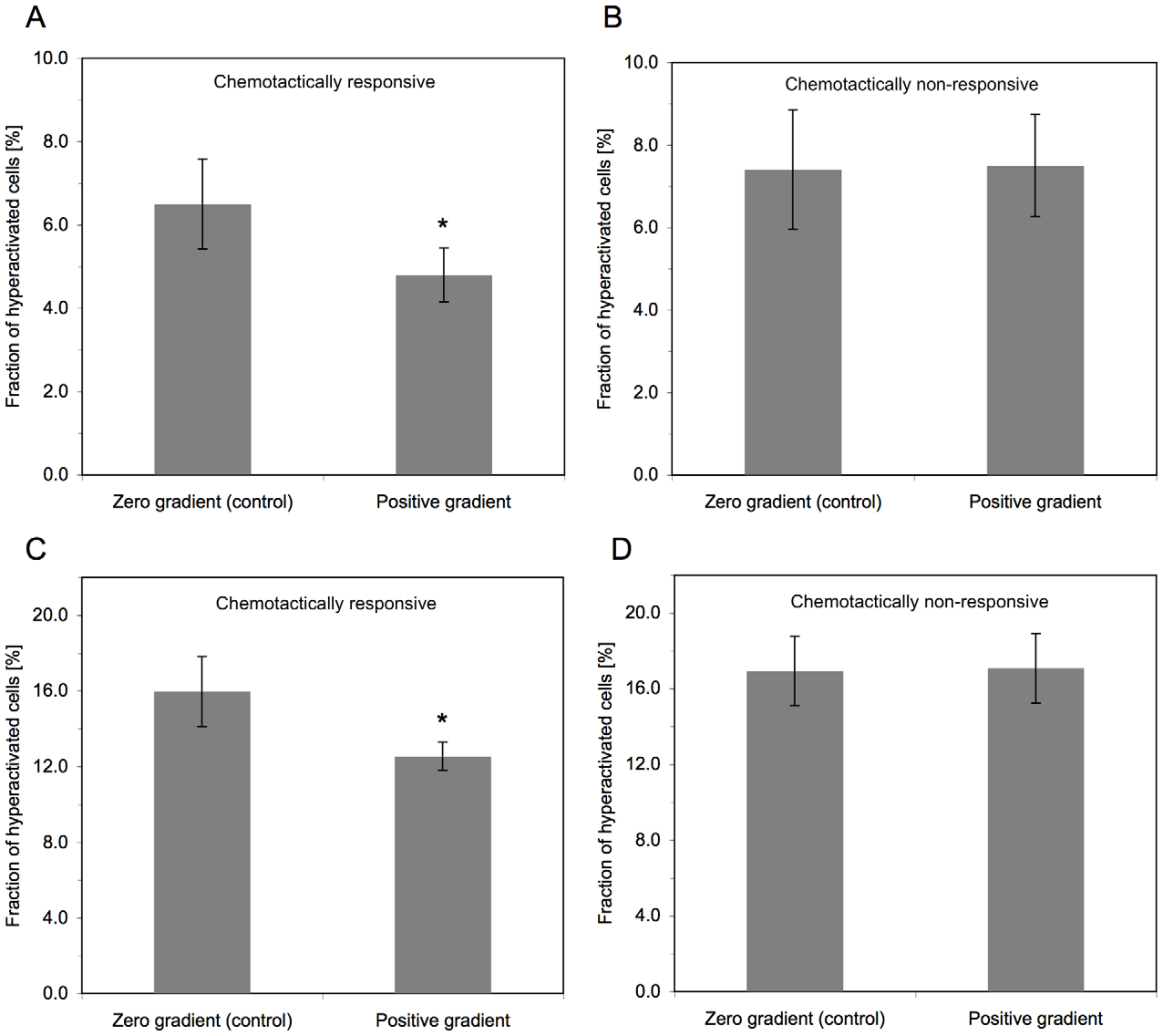
A comparison between the fractions of hyperactivated cells in chemotactically responsive and non-responsive spermatozoa. **A:** Sperm populations chemotactically responsive to progesterone (0.01–10 nM, depending on the sperm sample). The data shown are the mean±SEM of 20 or 40 runs (for the zero-gradient control or the positive gradient, respectively). **B**: Chemotactically non-responsive sperm populations in concentration gradients of progesterone similar to those in A. The definition of non-responsive population is explained in Materials and Methods. The data shown are the mean±SEM of 14 or 17 runs (for the zero-gradient control or the positive gradient, respectively). **C**: Sperm populations chemotactically responsive to progesterone (0.01–1 nM, depending on the sperm sample) in the presence of 5 mM procaine. The no-gradient control, as well, included procaine. The data shown are the mean±SEM of 6–7 runs. **D**: Chemotactically non-responsive sperm populations in concentration gradients of progesterone similar to those in C in the presence of 5 mM procaine. The no-gradient control included procaine. The data shown are the mean±SEM of 7–8 runs. In all cases, the no-gradient controls contained a gradient of DMSO (the solvent of progesterone) instead of a gradient of progesterone. An asterisk indicates a statistically significant difference from the control [*P* <0.05 (one-tailed) according to Mann-Whitney test in (A) and Student's t-test in (C)].

In the absence of a suitable inhibitor, we employed an indirect approach. We anticipated that if, indeed, hyperactivation is part of the chemotactic response and its role is to change the direction of swimming, the fraction of hyperactivated spermatozoa (reflecting the number of hyperactivation events because the identification of a cell as hyperactivated is based on the average behavior along the whole track) would be lower in a chemoattractant gradient, where capacitated spermatozoa would maintain their course of swimming once they find the right direction (up the gradient). However, since human spermatozoa swim in rather straight lines and the fraction of hyperactivated spermatozoa is low [27], the extent of expected change in hyperactivation level would be small. We compared the hyperactivation levels in chemotaxis experiments between two types of sperm population: those that, while in the chemoattractant gradient, reacted to the gradient by aligning their swimming direction with the gradient's direction (defined as chemotactically responsive), and those in which a chemotactic response could not be detected (defined as chemotactically non-responsive; see Materials and Methods for explanations). As controls, we also compared these populations with runs of the same populations in the absence of a gradient. Clearly, hyperactivation in the chemotactically responsive populations was significantly lower than in the non-responsive populations and the controls (Figure 1A, B). The use of progesterone as a chemoattractant strongly suggests that this reduction in hyperactivation events is linked to chemotaxis and not to another effect of progesterone. This is because progesterone is known to elevate hyperactivation [28], whereas the effect observed here is reduction of hyperactivation in spite of the presence of progesterone.

**Figure 2:**
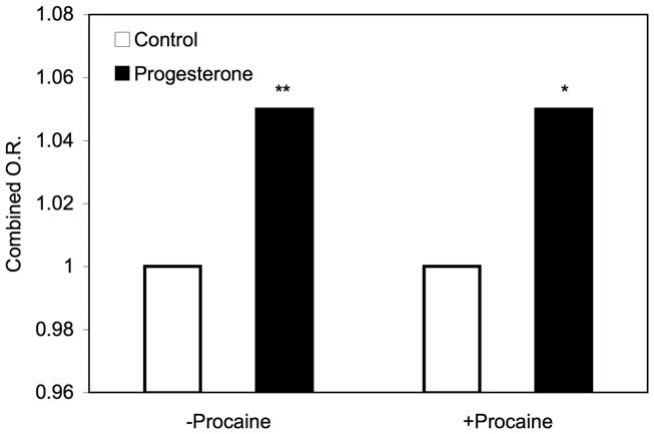
Chemotactic response of human spermatozoa to progesterone in the presence and absence of procaine. The progesterone and procaine concentrations were 0.01–1 nM (depending on the sperm sample) and 5 mM, respectively. The chemotaxis assays were carried out and analyzed as described in Materials and Methods. The intensity of the chemotactic response is reflected in the combined odds ratio (O.R.; defined in Materials and Methods); this parameter is close to 1 when cells swim randomly, and >1 when they swim preferentially in the gradient direction. The negative control is defined as O.R. = 1. Note that error bars are missing because the combined O.R. value is calculated from a pool of the whole data and is not an average. In addition, the use of the standard formula for confidence intervals for odds ratios is inappropriate in this case due to existence of correlations between the data points of the same sperm track (Armon *et al*., submitted). *, *P* = 0.06; **, *P* = 0.03.

The significant difference observed in the above experiments led us to make the same comparison also at elevated hyperactivation levels, thereby increasing the measurement range. This was achieved by including in the suspension procaine, known to elevate the fraction of hyperactivated spermatozoa in sperm [29], [30]. Procaine did not affect the chemotactic response to progesterone (Figure 2), even though it significantly elevated the hyperactivation level from 6.5±1.1% to 16.0±1.9% (±SEM; n = 20 without procaine and n = 6 with procaine; *P* = 0.0015, two-tailed Mann-Whitney test). Here, too, the fraction of hyperactivated spermatozoa in the chemotactically responsive runs was significantly lower than in the non-responsive runs and in the controls (Figure 1C, D).

The fact that a reduced number of hyperactivation events was only observed in experimental runs in which a chemotactic response was detected suggests that hyperactivation is part of the chemotactic response and that its role is to change the swimming direction. (However, not every turn must be the outcome of a hyperactivation event. Just like in bacterial swimming and chemotaxis [31], spontaneous turns or turns in response to subtle stimulation may well be gradual and moderate.) In other words, chemotactically responsive human spermatozoa in a spatial chemoattractant gradient seem to swim more linearly, exhibiting fewer hyperactivation events.

**Figure 3:**
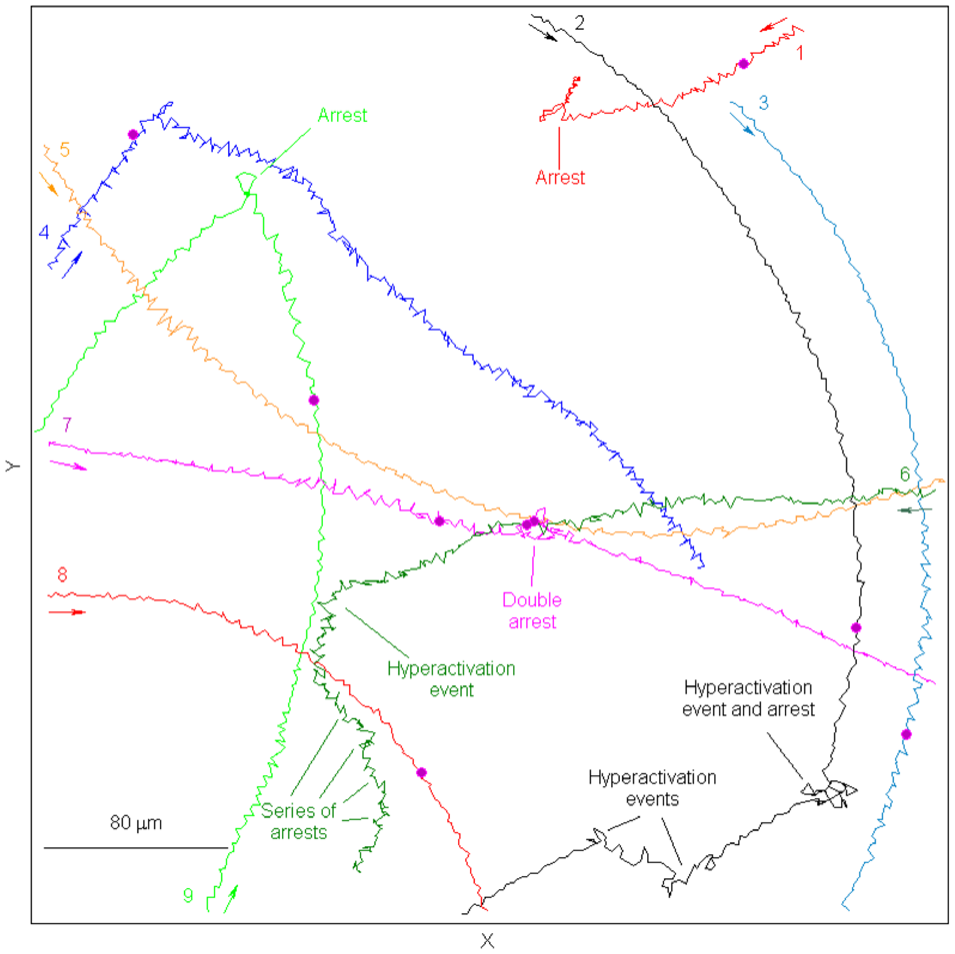
Tracks of spermatozoa showing different types of responses to photorelease of progesterone. The arrows indicate the direction of swimming. The purple dot indicates the time of the flash.

**Figure 4 pone-0028359-g004:**
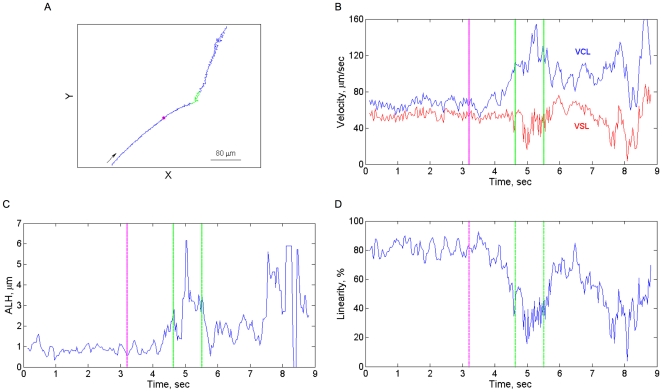
A representative swimming response, involving a hyperactivation event and a turn, to photoreleased progesterone. **A:** The trajectory made by the cell, monitored at 30 frames/s. The arrow indicates the direction of swimming. **B–D:** Kinematic parameters calculated from the trajectory. The calculations were done as described in Materials and Methods. The pink color indicates the time of the flash; the green color highlights the turn segment.

**Figure 5:**
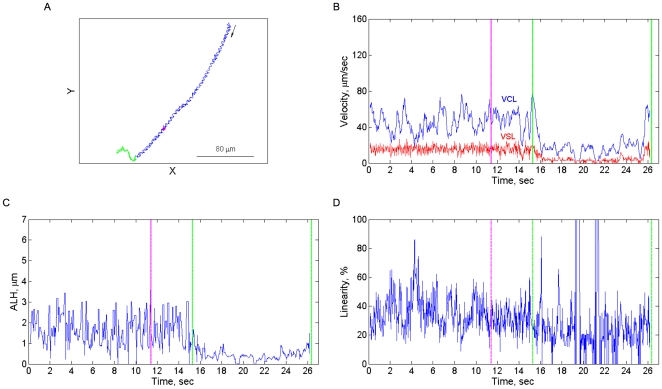
A representative swimming response, involving a flagellar arrest, to photoreleased progesterone. **A:** The trajectory made by the cell, monitored at 30 frames/s. The arrow indicates the direction of swimming. **B–D:** Kinematic parameters calculated from the trajectory. The calculations were done as described in Materials and Methods. The pink color indicates the time of the flash; the green color highlights the turn segment.

Wishing to examine this conclusion at the single-cell level, we carried out flash experiments in which progesterone was photoreleased in a sperm suspension. Generally speaking, the responses were similar to those observed earlier with cAMP or cGMP photorelease [22], though the fraction of cells responding to progesterone was lower (37%) (see Movie S1 in Supplementary Information for a typical example of the sperm response to photorelease of progesterone; see Figure 3 for the tracks made by the spermatozoa in this movie). The common denominator of all the responsive cells was a delayed turn, the length of the delay being 2.2±0.3 s (±SEM) on average. The differences between the responses were in the intensity of the response. Thus, of 75 sperm tracks analyzed, 10 cells (13%) responded to the flash with a delayed hyperactivation event resulting in a change of the swimming direction (Figure 4 as an example; cells #2 and #6 in Figure 3). In two of these cells the hyperactivation was immediately followed by an arrest episode (e.g., cell #2 in Figure 3). Additional 14 cells (19%) responded to the flash with a delayed flagellar arrest (Figure 5 as an example; cells #1, #7 and #9 in Figure 3; the arrest, 2–10 s in length, probably reflects a very intensive response). Whenever we could measure, the arrest was followed by swimming in a new direction (10 cells; in the other 4 cells the track recording ended before the resumption of the swimming). Noteworthy, some of the cells responded by more than one hyperactivation or arrest events (e.g., cells #2 and #6, respectively, in Figure 3). Other 4 cells (5%) responded with a delayed change in their swimming direction without a hyperactivation event (e.g., cell #4 in Figure 3 for a sharp turn). The rest of the cells (63%; cells #3, #5 and #8 in Figure 3) did not exhibit a detectable change in the swimming direction. The observed link between hyperactivation and changes in swimming track following the photorelease of progesterone endorses the notion of hyperactivation being part of the chemotactic response to chemoattracts (see Discussion).

## Discussion

The results of this study suggest that the rather erratic motion, known as sperm hyperactivation, is part of the chemotactic response of human spermatozoa. This conclusion is based on the reduction of hyperactivation events in chemotactically responding sperm populations in a spatial chemoattractant gradient (Figure 1), on the observation that, in response to a temporal concentration jump of a chemoattractant, turns often involve hyperactivation events (Figure 3), and on the published observations that both sperm hyperactivation and taxis are restricted to capacitated cells [3], [20], [21]. This conclusion is further endorsed by the recent findings that the CatSper channel (or a protein closely associated with it), well known to be involved in hyperactivation [10], is the receptor for the chemoattractant progesterone [14], [15]. Below we discuss the implications of this study to sperm behavior in a spatial chemoattractant gradient and we address a number of apparent difficulties and questions.

### Sperm response to a temporal chemoattractant gradient

Even though the photorelease of progesterone triggered a variety of different responses, they all seemed to share a common denominator: a delayed change in the swimming direction (Figures 3–5). It seems reasonable that the differences between the responses were more a matter of intensity than a matter of distinct response types. Thus, different cells may be differently sensitive to changes in progesterone concentration (e.g., due to differences in the number of receptor molecules in the membrane). These differences in sensitivity may be reflected in different responses to the extremely strong stimulus: from a delicate response (e.g., subtle turning), through a strong response (e.g., a series of hyperactivation events), to a very intense response (e.g., flagellar arrest in hypersensitive cells).

It is important to emphasize that in the progesterone photorelease experiments, as in any assay that measures the response of cells to a temporal chemoattractant gradient, the stimuli were orders of magnitude stronger (concentration-wise) than those encountered in a spatial gradient. This is a necessity due to differences in the detection threshold. A single small concentration change of the stimulant in a temporal assay may be undetectable whereas the same repeated change in a spatial gradient may be detectable due to the integration of the response over time [31]. In this study progesterone was photoreleased from its caged compound within ∼60 ms, reaching a concentration of 50 nM. This means that the photorelease created a temporal gradient of ∼1 µM/s. This gradient is 2–3 orders of magnitude steeper than the gradient in chemotaxis assays *in vitro* or the gradient established by the release of progesterone from cumulus cells, because progesterone is sensed at 0.1 pM ([32] and Figure 2) by spermatozoa that swim at an average speed of 70 µm/s, meaning a gradient at the order of 7 nM/s.

### Comparison between the sperm responses to photorelease of progesterone and cyclic nucleotides

The responses to photorelease of progesterone and their diversity are very similar to those observed in response to the intracellular photorelease of cyclic nucleotides [22]. There is, however, one major difference. In the case of the response to intracellular cAMP and cGMP, the large majority of the cells responded [22], whereas here, in response to progesterone, only about one third of the cells responded. This difference likely has two causes. First, the concentrations of cyclic nucleotides were orders of magnitude higher than those of progesterone. Second, one of the factors that probably determines whether or not a spermatozoon would respond to a chemoattractant is its sensitivity to the chemoattractant [22], e.g., the relative number of chemoreceptor molecules exposed to the medium. The response to cAMP or cGMP inside the cell is a post-chemoreceptor step; it is, therefore, independent of the chemoreceptors level and it is non-restricted by it.

### Apparent difficulties and questions

#### Aren't the responses to temporal and spatial stimulations of progesterone conflicting?

The response to progesterone stimulation in a temporal gradient (progesterone photorelease) was increased hyperactivation (Figure 3), whereas the response to progesterone in a spatial gradient was decreased hyperactivation (Figure 1). These observations are not conflicting, as they may seem. The difference between the observations is due to fact that the response in the temporal gradient consists of two phases: a delay followed by a hyperactivation/turn event(s) (Figure 3). Therefore, the continuous stimulation in the spatial gradient results in observing the first phase only (see below), meaning a reduced level of hyperactivation.

#### How could the fraction of cells responding to progesterone be higher than the fraction of capacitated cells?

It is well established that only capacitated cells are chemotactically responsive [3], [33], [34]. Nevertheless, the fraction of cells responding to progesterone in the photorelease experiments was higher than the fraction of capacitated cells. This apparent conflict is resolved by the difference between temporal and spatial gradients. Thus, in a spatial chemoattractant gradient, where the stimuli are always mild, only capacitated spermatozoa respond [3]. However, in response to a very strong stimulation as in the case of a concentration jump of photoreleased progesterone, even non-capacitated cells, which are presumably less sensitive to the chemoattractant [22], can respond. This possibility is well in line with the recent observation that in a capacitated sperm population the CatSper channel is more sensitive to activation by progesterone [14], [15] (possibly reflecting the higher number of exposed receptor molecules in capacitated cells).

#### Are sperm hyperactivation and bacterial tumbling similar phenomena?

Even though both hyperactivation and tumbling appear to serve the same function, i.e., abrupt change in the swimming direction, they are not the same. Tumbling, unlike hyperactivation, occurs in response to negative stimulation (increased concentration of a chemorepellent or decreased concentration of a chemoattractant). Hence, in bacteria like *E. coli*, photorelease of a repellent results in instantaneous tumbling whereas photorelease of a chemoattractant results in swimming linearization [35]. In contrast, in sperm cells, hyperactivation occurs in response to chemoattractant stimulation and it is preceded by a delay. Thus, both phenomena are mechanistically different but they apparently serve similar functions.

#### How procaine does not interfere with the chemotactic response?

The conclusion that hyperactivation is part of the chemotactic response raises the question of how procaine, shown to elevate the hyperactivation level [29], [30] (Figure 1), does not interfere with this response (Figure 2). Two reasons may account for this. First, procaine caused partial elevation of hyperactivation, from 6–7% to 16–17% (Figure 1). This leaves sufficient room both up and down for chemotaxis-dependent modulation of hyperactivation events. In analogy, it is well known that the tumbling levels of different bacterial populations may differ from each other and, yet, the populations may be chemotactically responsive to a similar extent. Second, the chemotactic response is carried out by capacitated cells only [3], [33], [34] whereas the procaine effect is not selective [36]. Therefore, in the presence of procaine the fraction of capacitated cells (i.e., the fraction of the chemotactically responsive cells) may be as high as in the absence of procaine.

### Implications to sperm behavior in a spatial chemoattractant gradient

**Figure 6:**
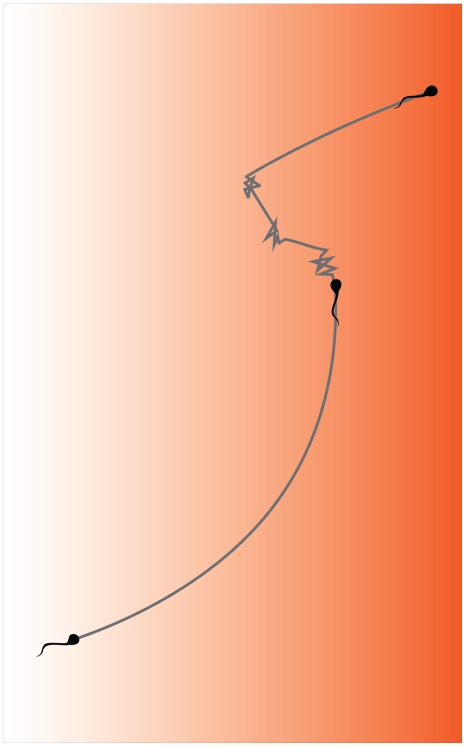
A model for the behavior of human spermatozoa in a spatial chemoattractant gradient. The intensity of the background color represents the chemoattractant concentration. As long as the cell senses an increase in the chemoattractant concentration, turns and hyperactivation events are repressed, and the cell swims in a roughly straight line. However, when the cell ceases sensing an increase in the chemoattractant concentration, it turns and executes hyperactivation episodes, as a result of which its direction of swimming changes. This may happen over and over until the cell happens to swim up the concentration gradient again (or until it senses no concentration change, in which case it will eventually adapt and return to the non-stimulated swimming mode — not shown in the figure).

Earlier, the response of human spermatozoa to a concentration jump in the intracellular concentration of cyclic nucleotides combined with the response to manual addition of chemoattractants led us to propose a model for the behavior of human spermatozoa in a spatial chemoattractant gradient [22]. The results of the current study and the similarity between the responses of the cells to photorelease of progesterone and cyclic nucleotides not only endorse our proposed model but they also expand it to include hyperactivation (Figure 6). According to the expanded model, when a capacitated spermatozoon swims up a concentration gradient, it is continuously stimulated, resulting in suppression of turns and hyperactivation events. The observed reduced level of hyperactivation during a response to a spatial chemoattractant gradient (Figure 1) is fully consistent with the model. When the spermatozoon swims down the gradient or when it senses no change in the chemoattractant concentration it exhibits turns and hyperactivation episodes to modify its direction of swimming. In the case of sensing no change, the cell adapts to the constant chemoattractant concentration and restores its non-stimulated swimming mode, consisting of rather straight swimming with occasional hyperactivation events and turns.

Spermatozoa exhibit two phases of response to the chemoattractant, a delay and a turn (or series of turns; Figures 3–5). According to the model, the first phase is dominant in an upward chemoattractant gradient, and the second phase is dominant in a downward gradient or when the upward gradient is not sensed anymore. Two mechanisms may account for the two-phase response. One is that the two phases are independent of each other; the first phase occurs when the chemoattractant concentration increases, and the second phase occurs when no change in the concentration is sensed or when the chemoattractant concentration decreases. The other possibility is that both phases are linked, being an integral part of the very same response. The available data cannot distinguish between these possibilities.

## Materials and Methods

### Ethics statement

The study was approved by the Bioethics and Embryonic Stem Cell Research Oversight Committee of the Weizmann Institute of Science. Informed consent was obtained in writing from each sperm donor.

### Spermatozoa

Human semen samples were obtained from ten healthy donors after 3 days of sexual abstinence. Semen samples with normal sperm density, motility and morphology (according to WHO guidelines [37]) were allowed to liquefy for 30–60 min at room temperature. Human spermatozoa were separated from the seminal plasma by the migration–sedimentation technique [38] using the commercially available Modified HTF medium (Irvine Scientific, Santa Ana, CA, USA) supplemented with 0.3% human serum albumin (HSA, Irvine Scientific, Santa Ana, CA, USA). Following this procedure, the sperm concentration was adjusted to 4×10^5^ cells/ml in HTF medium containing 0.3% HSA and 3.5% polyvinylpyrrolidone (PVP 25 K, Fluka, Buchs, Switzerland). PVP was used for two reasons. One was to elevate the viscosity of the medium, making it closer to the physiological environment *in vivo* [39], [40]. The second reason was that PVP is a hydrophobic carrier [41], thus preventing the adsorption of the hydrophobic chemoattractants to the chemotaxis chamber. The sperm suspensions were incubated under an atmosphere of 5% CO_2_ at 37°C for an additional 1 h (in total, 2 h together with the separation procedure) to obtain capacitated spermatozoa [3].

### Chemotaxis assays

Chemotaxis assays were performed at room temperature in a disposable µ-slide chemotaxis, consisting of two reservoirs (40 µl each) connected by a thin slit (2×1×0.07 mm; Ibidi GmBH, Munich, Germany). Sperm suspensions and chemoattractant solutions were adjusted to room temperature prior to the experiment. When used, procaine (Sigma-Aldrich, Steinheim, Germany) was added just prior to chamber loading. Next both reservoirs and the slit were filled with spermatozoa, and only then the chemoattractant was applied within a sperm suspension (in order to avoid dilution of the cell concentration) to one of the reservoirs. In control experiments, cell suspension containing the solvent of the chemoattractant was applied instead. After loading, the slides were incubated at room temperature for 20 min to allow the establishment of a chemoattractant concentration gradient (Figure S1 in Supplementary Information). The swimming of spermatozoa in the observation area (part of the slit) was video-recorded for 4 min at two different fields (changed every 40 s) using Nikon Eclipse Ti microscope at 10x magnification. The cell tracks (10–450 µm long) were subsequently analyzed with homemade software.

### Chemotaxis assessment and statistical analysis

Chemotaxis was evaluated as described [42] on the basis of distribution of the instantaneous directionality angles (γ_inst_, the angle between the vector of the cell frame-to-frame displacement and the gradient direction; the video frequency was 25 frames/s). For each treatment the total number of angles in the gradient direction (N_+_) and in the opposite direction (N_-_) were summed from a number of experiments. A combined odds parameter was calculated as the ratio between these sums (combined Odds = ΣN_+_/ΣN_-_). The odds parameter yields values close to 1 when the swimming is random; it is >1 when the swimming is biased in the gradient direction. The intensity of the chemotactic response was reflected in the combined odds ratio (O.R.) parameter (combined O.R. = combined Odds_treatment_/combined Odds_control_). The statistical significance of the response was estimated by adjusting the distribution of combined O.R. values using bootstrapping algorithm that sub-samples all the angles corresponding to a random selection of control tracks, as described in Armon *et al*. (submitted).

### Analysis of hyperactivation

All recorded sperm tracks of chemotaxis assays were re-analyzed by the computerized motion analysis system to determine the fraction of hyperactivated spermatozoa in them. Those runs that yielded a positive response (O.R. >1.02) were defined as the responsive population. Chemotaxis assays of human spermatozoa are characterized by a very low signal-to-noise ratio — an outcome of the small fraction of responsive cells [43], [44], [45], i.e., of capacitated cells [2], [3]. Consequently, in some runs of the assay (any type of a chemotaxis assay) the spermatozoa are similarly distributed in all directions and chemotactic activity is not detected, even though the same chemoattractant concentration is used. These runs were considered as chemotactically non-responsive. Among the non-responsive runs only those having the same concentration of progesterone (or one order of magnitude higher) as the responsive runs of the same experiment were taken into account. The determination of whether a cell is hyperactivated or not was done on the basis of its curvilinear velocity (VCL; mean velocity of the sperm head along its actual, sampled path [46], [47]) and fractal dimension (FD; an indicator of the sperm trajectory regularity (describing its space-filling properties) [48]. Cells having VCL >70 µm/s and FD >1.7 were defined as hyperactive. This definition is based on the study by Mortimer *et al*. [48], which determined the value of FD >1.3 as representing hyperactivated tracks, irrespectively of the sampling frequency. To restrict our analysis to fully hyperactivated cells (not transitional), we elevated the threshold to FD >1.7. VCL was used as an additional parameter, in accordance with Mortimer *et al*. [48]. The percentage of hyperactivated cells was calculated out of the motile population only. It should be noted that because the VCL and FD values are averaged over the whole track, a cell that exhibits isolated brief hyperactivation events would likely not be considered as hyperactivated by the motion analysis software. The statistical analysis was carried out using InStat 3 software (Graph Pad Software, San Diego, CA, USA).

### Flash-photolysis

Sperm cells were mixed with 500 nM caged progesterone [49] and placed into an observation chamber (50 µm depth). Motility of sperm cells was recorded with an inverted microscope (IX71; Olympus, Hamburg, Germany) equipped with a 20x magnification objective (UPLSAPO; NA 0.5; Olympus). Experiments were carried our in a 37°C incubator (Life Imaging Services, Basel, Switzerland). Photolysis of caged progesterone was achieved using a 100 W Mercury lamp (U-RFL-T; Olympus). Light was filtered through a band-pass filter (H 350/50; AHF analysentechnik, Tübingen, Germany). The irradiation time (∼60 ms) was controlled by a mechanical shutter (VS25; Uniblitz, Vincent Associates, Rochester, USA). Under the experimental conditions used, the energy density at the focal plane was 619 mJ/cm^2^ and ∼10% (equivalent to 50 nM) of the total caged progesterone was expected to be released per flash. To produce sharp images of swimming sperm, stroboscopic illumination was achieved using a white LED (K2 star; Luxeon) and a custom-made housing. Illumination pulses (2 ms) were triggered using a programmable waveform generator (33220A; Agilent, Böblingen, Germany). Images were collected at 30 frames/s using a back-illuminated EMCCD camera (DU-897D; Andor, Belfast, Northern Ireland).

### Analysis of the sperm response to progesterone photorelease

The analysis of sperm behavior was carried out by homemade software, which provides track coordinates and commonly used kinematic parameters of individual cells, calculated as a moving average with a 9 frames window. These kinematic parameters include the straight-line velocity (VSL; defined as the time-average velocity of the sperm head along a straight-line from its first position to its last position), VCL, the linearity of swimming path (LIN; the ratio VSL/VCL), and the amplitude of lateral head displacement (ALH; the amplitude of the variations of the actual sperm-head trajectory around its average path) [46], [47]. The ALH was calculated as twice the local maximal distance from the true track coordinates to their corresponding averaged coordinates [47].

## Supporting Information

Figure S1 **Gradient establishment in the** µ**-slide chemotaxis chamber.** Rhodamine B was used instead of chemoattractant and the fluorescence intensity measured as a function of the distance from the chemoattractant reservoir. The region shown is the recording area, which is only a part of the whole observation area.
(TIF)

Movie S1 **Sperm response to photorelease of progesterone.** Spermatozoa showing different types of responses to photorelease of progesterone, monitored at 30 frames/s. The flash for progesterone photorelease was at 5 s.
(MOV)
