# Supplementary figures and images for: Behavioral Mechanism during Human Sperm Chemotaxis: Involvement of Hyperactivation
Source: PLoS One. 2011 Dec 7;6(12):e28359. doi: 10.1371/journal.pone.0028359 (PMC3233563; doi:10.1371/journal.pone.0028359)

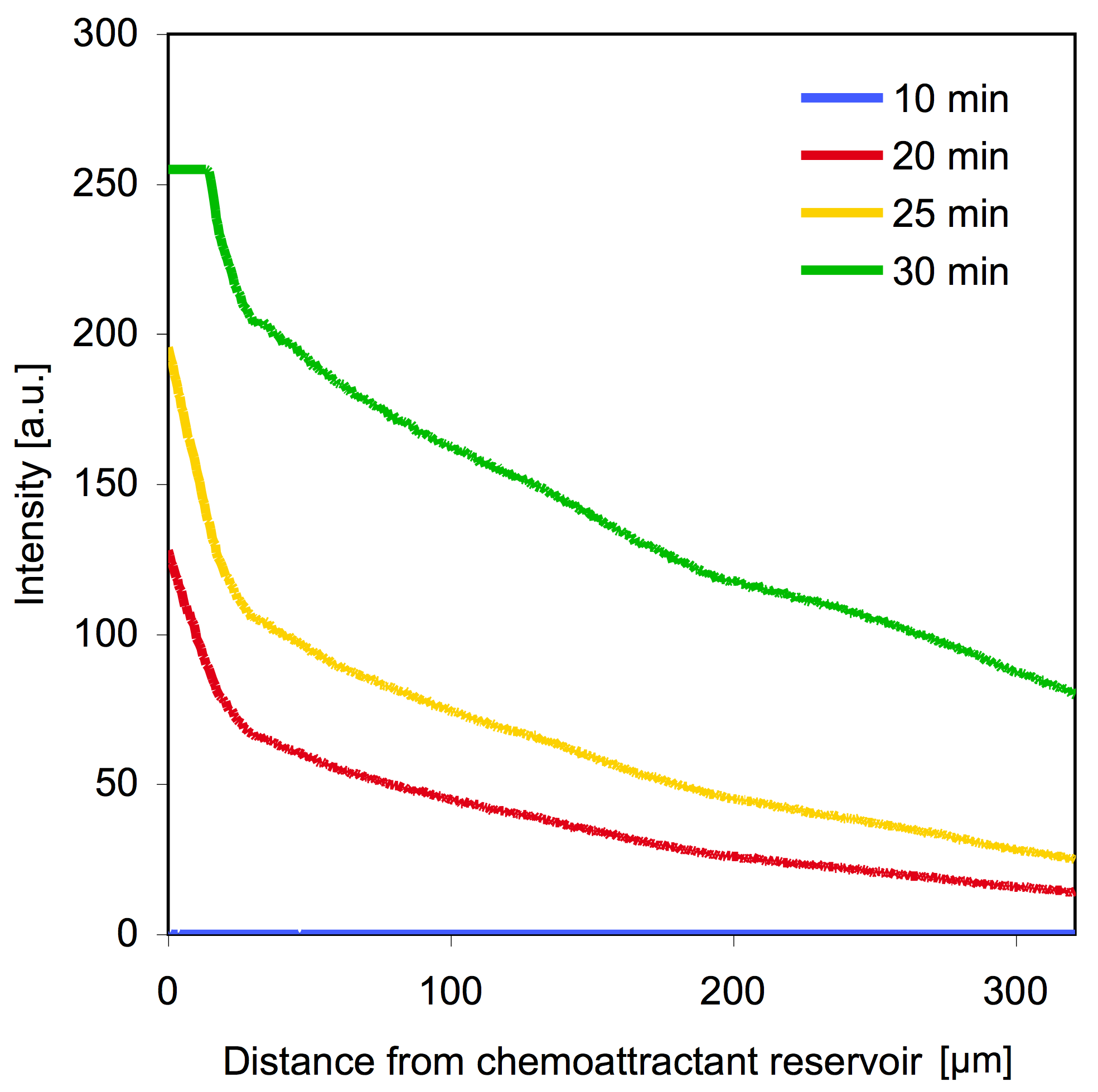

Supplement: Figure S1 — Gradient establishment in the µ-slide chemotaxis chamber. Rhodamine B was used instead of chemoattractant and the fluorescence intensity measured as a function of the distance from the chemoattractant reservoir. The region shown is the recording area, which is only a part of the whole observation area. (TIF) [file pone.0028359.s001.tif]
